# Barriers and enablers to testing for hepatitis C virus infection in people who inject drugs – a scoping review of the qualitative evidence

**DOI:** 10.1186/s12889-023-16017-8

**Published:** 2023-06-01

**Authors:** Cathy R. Balsom, Alison Farrell, Deborah V. Kelly

**Affiliations:** 1 School of Pharmacy, Memorial University of Newfoundland, St. John’s, Canada; 2 Health Sciences Library, Memorial University of Newfoundland, St. John’s, Canada

**Keywords:** Hepatitis C Virus, Injection Drug Use, Testing, Public Health

## Abstract

**Background:**

Injection drug use is the primary mode of transmission of hepatitis C virus (HCV) infection in the developed world and guidelines recommend screening individuals with current or history of injection drug use for HCV; however, the majority of those living with HCV in Canada are not aware of their positive status. This low level of HCV status awareness suggests that screening is not effective with current testing strategies. The aim of this review is to determine what barriers and enablers people who inject drugs (PWID) experience surrounding testing for HCV to help inform the development of an engaging testing strategy.

**Methods:**

Comprehensive literature searches were conducted using Medline, Embase and CINAHL in February 2021. Included studies investigated the barriers and enablers to testing for HCV in PWID and the experiences of PWID in testing for HCV. Studies were included if they were qualitative or mixed-methods design, involved people with current injection drug use or those with a history of injecting drugs, and were written in the English language. Studies were compared and common themes were coded and analyzed.

**Results:**

The literature search resulted in 1554 citations and ultimately nine studies were included. Common barriers included self-perception of low risk for HCV, fear of diagnosis, stigma associated with IV drug use and HCV, antipathy in relation to mainstream health care services, limited knowledge about HCV, lack of rapport with provider, lack of motivation or competing priority of drug use, and limited awareness of new treatment options. Common enablers to testing included increasing awareness of HCV testing and treatment and providing positive narratives around HCV care, positive rapport with provider, accessible testing options and individualized care.

**Conclusion:**

While there has been some qualitative research on barriers and enablers to testing for HCV in PWID more research is needed to focus on this research question as a primary objective in order to provide more understanding from the participant’s perspective.

**Supplementary Information:**

The online version contains supplementary material available at 10.1186/s12889-023-16017-8.

## Background

Globally, an estimated 58 million people have chronic hepatitis C virus (HCV) infection and there are 1.5 million new infections each year [1], however, many people living with this infection are unaware they have it. The World Health Organization (WHO) states that only 19% of people living with HCV are aware of their infection [2], leaving 81% unaware. HCV occurs in all regions of the world and although the Americas have the lowest prevalence worldwide, an estimated 5 million people in this region live with HCV infection yet only 22% have been diagnosed [2]. In 2016, the WHO called on all countries to invest in eliminating hepatitis and has set a goal of eliminating viral hepatitis by 2030, unfortunately many experts say that without increasing awareness, expanding testing, and linking patients to care it is unlikely this goal will be met [3].

HCV is a blood borne virus; infection occurs through exposure to blood containing virus through unsafe injection practices, unsafe healthcare practices, injection drug use and unprotected sexual practices that lead to an exposure to blood [1]. Once infected with HCV, symptoms can take 2–6 months to appear, and the majority of cases are asymptomatic but the infection can still be spread during this time. 70% of those infected with HCV will develop chronic infection which can lead to liver fibrosis, cirrhosis, end-stage liver disease, and hepatocellular carcinoma [1].

Injection drug use is the primary mode of transmission of HCV infection in the developed world and guidelines recommend screening individuals with current or history of injection drug use for HCV [4]; however, the percentage of people who inject drugs (PWID) who are unaware of their HCV infection in Canada has been reported to be as high as 70% [5]. The low level of HCV status awareness suggests that screening in this population is not effective with current strategies.

There have been major advancements in the treatment of HCV in the past decade with the introduction of direct-acting antiviral (DAA) therapies [6] which are more effective, safe, and better tolerated, leading to increased cure rates of HCV infection [7]. If people are unaware of their HCV infection and thus not linked to care, they will not be able to realize the advantages of these agents. Treatment prevents disease progression, limits future morbidity and mortality and prevents cirrhosis, liver transplantations and liver cancer [8], but people would need to be aware of their infection to be linked to care. Understanding their barriers to testing is necessary to inform development of engaging testing options for PWID. This is the first step in improving health outcomes, as well as decreasing transmission and achieving viral eradication.

This qualitative scoping review aimed to determine the barriers and enablers and experiences of PWID with regards to HCV testing. This knowledge could help guide the design of an HCV testing strategy that would engage PWID and increase HCV awareness and linkage to care.

## Methods

### Inclusion criteria

A search was conducted to identify studies which examined barriers and enablers to HCV testing among people who engaged in current injection drug use or had a history of injection drug use. Studies were included if barriers or enablers to HCV testing among PWID were addressed as either the primary or secondary outcome. Studies could be qualitative or mixed-methods provided that sufficient detail was included to permit understanding of the barriers and enablers. Only those studies which explored the experiences and perceptions of PWID were included. The search was not limited by age as injection drug use is a risk regardless of age. Only full-text publications reported in English were included.

### Exclusion criteria

Studies that did not address barriers or enablers to testing for HCV, and those that only included quantitative survey data were excluded. Studies which took place within prison settings were also excluded as the testing options and influences on accepting testing are expected to be different for people in prison in than those in the community.

### Search strategy

A librarian conducted comprehensive literature searches in Medline (via Ovid), Embase (embase.com) and CINAHL (via Ebsco) and included all studies prior to February 2021. The search was peer-reviewed by a second librarian using the Peer Review of Electronic Search Strategies checklist [9]. The final search strategy can be found in Additional file 1.

### Study selection process

Search results were imported into Covidence, an online primary screening and data extraction tool [6]. Two levels of screening were performed to identify articles for inclusion. Level 1 was based on a review of abstracts for relevance, and level 2 involved full-text review to ensure articles met inclusion criteria. The inclusion criteria were imported into the software and used by the reviewers during level 1 screening when considering titles and abstracts and during level 2 screening when completing the full-text review. Two reviewers, one author (CB) and one student (JK) independently completed both level 1 and level 2 screening, and discrepancies were reconciled by a third reviewer (DK).

### Synthesis

Data were charted to compare study designs and descriptions of barriers and enablers which repeated across studies (Tables 2 and 3). Coding of the results/findings section of each included study was done line by line by two reviewers (CB and AD) who independently reviewed each study to identify barriers and enablers associated with testing. Thematic analysis was completed by CB and codes were organized into themes based on how they related across studies. A final list of themes was agreed upon by DK and CB.

## Results

**Fig. 1:**
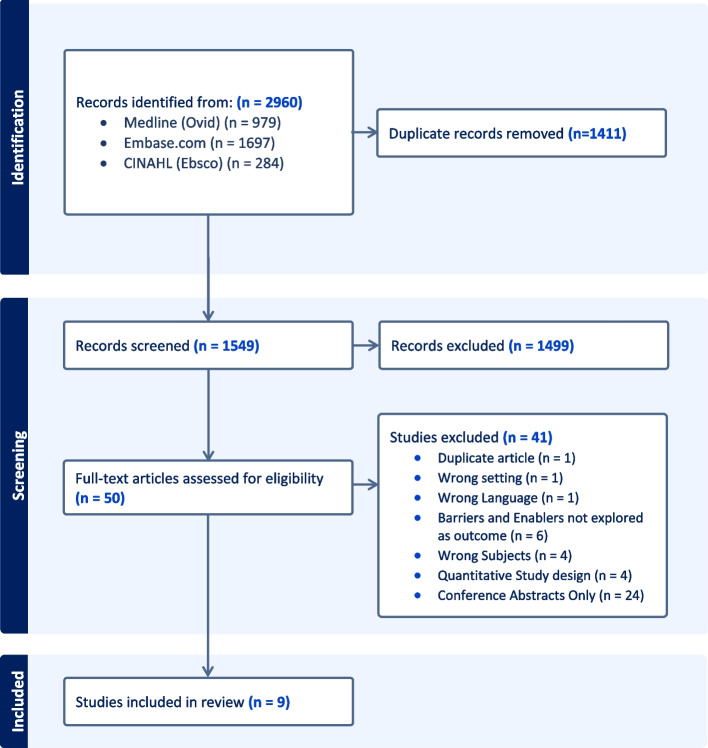
Study selection for the scoping review

The literature search resulted in 1554 citations (Fig. 1). After screening titles and abstracts, 50 studies were selected for full-text screening. Nine studies fulfilled the eligibility criteria and were included [10–18].

Most studies involved interviews except for one which used open ended questions at the end of a survey [16]. All reports were relatively recent, published within the last nine years (Table 1). Studies were set in the United States, United Kingdom or Australia. As the included studies were qualitative in nature, they tended to have a small sample size; however the survey involved 362 respondents [16] and another study involved 48 participants [11].

**Table 1:** Study characteristics

Item (*n* = 9)		Count
Year of publication	2014–2015	2
	2016–2017	0
	2018–2019	5
	2019–2020	1
	2021	1
Country	United States	4
	United Kingdom	3
	Australia	2
Setting	Syringe exchange program	2
	Community based outreach program	3
	Drug treatment service program	3
	Inpatient detoxification	1
Methods	interviews	8
	open-ended survey	1
Sample Size	10–19	2
	20–29	4
	30–39	1
	40–49	1
	50 +	1

Two studies examined participant experiences with screening or testing for HCV as their primary objective [10, 16], while two others looked at the whole care contiuum, from screening to treatment and monitoring [15, 17] (Table 2). The remaining studies looked at experiences of participants in specific testing programs [11, 13] or specific modes of testing delivery such as community-based outreach, point-of-care testing, and social network interventions [12, 14, 18]. The settings of the studies varied; athough multiple studies were set within the community (community outreach programs or needle syringe exchange programs), one was set in an inpatient detoxification setting. Three studies were set in drug treatment services programs or opioid substitution therapy prescribing services. Common barriers and enablers were seen across all studies (Table 3).

**Table 2:** Study Descriptions

Authors, Year, Country	Primary Objective	Inclusion Criteria	Setting	Participant Characteristics
Barocas et al., 2014 [16], United States	To examine characteristics associated with HCV screening among PWID utilizing a free, multi-site syringe exchange program and to elicit their perceptions of barriers and facilitators to screening for HCV	18 years of age or older, could speak and read English and reported a history of injecting drugs	Syringe exchange program operating through office-based locations in two large cities and via mobile van units that serve the suburbs, surrounding rural communitites, and other smaller cities in Wisconsin	69% male Median age of 28 83% white
Coupland et al., 2019 [12], Australia	To explore the impact of the Hepatitis C Incidence Transmission community-based outreach model in engaging and retaining newly diagnosed PWID in HCV testing as an entry point into the HCV care cascade	16 years of age and older, have injected drugs in the previous 12 months, and acquired HCV infection while enrolled in a community-based prospective observational study of hepatitis C vaccine preparedness	Community based outreach program in Sydney, Australia	57% male Median age of 27 36% self-reported a culturally diverse background
Harris et al., 2018 [11], UK	To inform and assess the HepCATT study	Drug treatment services clients who currently or previously injected illicit drugs. Aged 19–69	Drug treatment services program in England	79% male Age range 19–69
Harris et al., 2014 [10], UK	To examine participants’ accounts of HCV testing, especially regarding barriers to uptake	18 years of age or older, proficient in English, currently injecting an illicit drug (within the last 30 days), and having injected illicit drugs for at least six years	Community based opioid substitution therapy prescribing services and drug user networks in London, England	22 HCV antibody negative and 15 postitive 73% male Age range 23–57 76% were white British
Latham et al., 2019 [18], Australia	To explore the acceptability of point-of-care testing for PWID within the larger Rapid-EC pilot study	Ages 19–56 with a history of injecting drugs and participating in the Rapid-EC pilot study offering point-of-care testing	Needle syringe programs in Melbourne, Australia	74% male Age range 19–56, median 44
Phillips et al., 2021, UK	To explore the experiences of clients and staff involved in Project ITTREAT and assess the facilitators and barriers to a community-based HCV service	Drug and alcohol treatment service attendees with current/previous HCV infection and past or current injection drug use that had been offered and/or were engaged in the HCV service	Drug and alcohol treatment services in England	80% Male 100% white
Skeer et al., 2018 [17], United States	To provide a greater understanding of the perspectives and experiences of young PWID navigating the HCV care continuum, and in turn, to inform future treatment as prevention strategies	Currently injecting drugs or had injected drugs in the past but weren’t actively injecting, self-disclosed HCV positivity and between the ages of 15 and 30	Community outreach program in Boston Massachusetts	50% male Age range 22–30 87.5% Non-hispanic white
Tofighi et al., 2020 [15], United States	To identify the complex interplay of social and environmental factors that influence engagement along the HCV care continuum	18 years of age and older admitted to inpatient detoxification for opioid use disorder	Inpatient detoxification hospital in New York City. Though thie particpants were admitted at the time of the interview the questions probed participants experience in the community, not in the hospital	78% male Age range 21–62 (mean 44) 35% African-American, 26% Hispanic 65% diagnosed with HCV
Ward et al., 2021 [14], United States	To evaluate perceptions of social network interventions for HCV testing, linkage to care, and treatment. In particular, PWID willingess to perform the peer mentor role and to understand the barriers and facilitators of this approach	18 years of age or older, English speaking, HCV antibody positive and had a history of injection drug use	Community based research organizations and provider referral in Baltimore, Maryland	75% male Age range 32–65 (mean 51) 75% Black/African American

**Table 3:** Barriers and Enablers to HCV testing

(Authors), year (Country)	Sample	Method	Barriers	Enablers
Barocas et al., 2014 [16], United States	*n* = 362	Open-ended survey questions	Fear of positive test Low perceived risk Stigma associated with HCV and/or IVDU Lab characteristic Lack of access to transportation Time contraints Lack of knowledge of testing Cost Lack of access to MD/PCP Not having to take initiative Lack of rapport with provider Confidentiality Lack of motivation	Health concerns for self or others High perceived risk Lab characteristic Access to transportation Mobile testing center/SEP Adequate time Knowledge of testing Free testing Access to MD/PCP Not having to take initiative Rapport with provider Confidentiality Motivation
Coupland et al., 2019 [12], Australia	*n* = 28 at baseline, 25 at 12 months	Semi-structured interviews		Building trust and willingness to be tested for hepatitis C Making information about HCV testing and treatment salient
Harris et al., 2018 [11], UK	*n *= 48	In-depth interviews, focus groups and observations	Perceived lack of testing availability Perception of minimal HCV risk Fear of diagnosis and HCV stigma Confusion about the testing and treatment process Aversions to having a venous sample taken Concerns of interferon treatment Desire to move away from a drug user identity Limited psycho-social ‘stability’ Testing could be destabilising if it revealed them to be HCV positive Perception of GPs lack of interest in them and HCV more generally	Intervention timeliness: capitalising on stability and new treatments Personalized and flexible care HCV visability and communication structure Streamlined testing and treatment pathways
Harris et al., 2014 [10], UK	*n* = 37	Qualitative life history research	Fear in relation to diagnosis Apathy regarding mainstream health care services Optimism in relation to risk knowledge Confusion in relation to HCV testing and its consequences	
Latham et al., 2019 [18], Australia	*n* = 19	Semi-structured interview	People and place Rapidity of result return	People and place Method of specimen collection Rapidity of result return
Phillips et al., 2021, UK	*n* = 15	Semi-structured interviews	Lack of stability Stigma Negative discourse around testing and treatment	Trusting client-provider relationship HCV as part of recovery pathways Mitigation of prevous negative experiences of secondary care Positive narratives around HCV care
Skeer et al., 2018 [17], United States	*n* = 24	Interviews	Deservingness of HCV treatment and stigma Perceived lack of referral to HCV treatment or follow up Disincentives around HCV treatment for PWID Perception of need for treatment	
Tofighi et al., 2020 [15], United States	*n* = 23	Interviews	Limited knowledge of HCV Limited access to healhtcare providers addressing HCV Competing priority of use Benign perceptions of HCV infection Limited awareness of the availability of DAAs Cost Inability to locate or receive referrals for screening Physician preferences to not initiate HCV treatment	Accessibility of testing sites
Ward et al., 2021 [14], United States	*n* = 20	Semi-structured interviews	Drug use and lack of social support Challenges to providing peer support	Perception of HCV prevalence within networks Willingness to provide support in social networks

### Barriers to testing

#### Perceived low risk for HCV

In the earliest paper, Harris et al. (2014) [10] reported that the risk of HCV among participants was perceived to be minimal. For example, one participant in their study stated that, "through them years, yeah, I have used a spoon that maybe someone else has used on it, but I've never used dirty works. You know, nothing dirty". A later paper by Harris et al. (2018) [11] reported that there was a perception of minimal HCV risk due to relatively safe injection practices and/or lack of symptoms. Barocas et al. [16] noted that participants perceived their risk of HCV to be low based on never sharing needles, lack of symptoms and having received a negative test result in the past.

#### Fear of diagnosis

Fear of learning test results was a barrier to testing; participants in the study by Harris et al. (2014) stated: "I don't think I'd be strong enough in my mind if I did have it to handle it [HCV] and it could make me spiral worse out of control. That would be my reason", "Just the fright of it [HCV], if I did have it probably, I don’t know, the fright of it", "I'd rather not know" and "I'd rather die of ignorance at that time" [10]. Participants across multiple studies [10, 11, 16] indicated they were “scared of the result” with one participant in the study by Barocas et al. quoted as saying “I worry about Hep C more than HIV. I’m afraid of what the result might be” and another stating “I’m in denial. I don’t want to hear that I have it” [16].

#### Stigma

Stigma was a noted barrier in multiple studies [11, 13, 16, 17]. Participants in the study by Barocas et al. perceived stigma associated with both injection drug use and HCV infection to be obstacles to seeking HCV testing and used words such as “shame”, “embarassment”, and “taboo” when describing their experience. "People know that most of the time you get tested for Hep C because you’re an IV user. People judge you no matter what your results are. That’s the worst feeling ever" [16]. One participant in the paper by Ward et al. stated.

They need more local like clinics. Like in the hood, in the ghetto, in the slums. Not just in the nice part of town – because they look like shit, so they don’t really want to go out, you know, to a Johns Hopkins or to a University of Maryland where you’ve got to go downtown and you’ve got to see regular people, you know, and feel judged or feel like people are talking about you or watching you [14].

#### Antipathy in relation to mainstream health care services

Participants actively avoided mainstream healthcare services because they expected discriminatory treatment, posing a barrier for HCV testing. In the study by Harris et al. (2014) multiple participants expressed this barrier, one stated: “I've never felt comfortable with any GP because, in the past, when you've gone to a GP you say ‘I wonder if you can help me, I take heroin…’’(exclamation of horror) sorry, I don't deal with that here’” [10]. Another participant in the same study had never been tested despite reporting 17 years of daily injecting. They stated, “They said to go up to the hospital [for a HCV test], and it’s like, I couldn’t be bothered to go up the hospital” [10]. This reluctance may be due to shame and embarassment when having to show their scars; “If a doctor wanted to examine us and I’d roll my sleeves up and I’ve got track marks, it was embarrassing man, it was horrible” [10]. Poor venous access and prior negative experiences were reasons to avoid going to get tested in the hospital: “What’s the point of going to the hospital? Cause if I can’t find a vein, they definitely won’t be able to find a vein” [10].

#### Limited knowledge of HCV and testing

Participants expressed limited knowledge of HCV; in the study by Tofighi et al. one participant stated: “I don’t even really fully understand Hep C. You get that from needles?” [15]. Harris et al. (2014) included multiple quotes from participants describing the misconception that HCV transmission risk is equal between injecting and sexual practices:

I shared with him because he was never unfaithful to me"and "I know I haven't got it [HCV] anyway… [because] I've probably had less than five sexual partners in the last 10 years and of them none of them are users [10].

Some participants had the misconception that there was a vaccine for Hepatitis C; “I’ve had boosters for hep C. I’m not due anymore boosters now” [10]. In the paper by Ward et al. there was a perception amoung some participants that lack of identifiable symptoms means that HCV was not serious which limited engagement in testing and treatment [14].

#### Lack of access to and rapport with provider

Both lack of access to a medical doctor/primary care provider and a lack of rapport with the provider are barriers to HCV testing. Two of the included studies noted limited access to healthcare providers for HCV care as a barrier [10, 15] and Barocas et al. noted lack of rapport with provider as a barrier [16]. One participant in the study by Harris et al. (2014) stated "I had one doctor who tried, you know, ‘Oh you need to go and get tested, 'and like he'd frighten me then and I'd go and see another doctor next time" [10]. Skeer et al. also noted this as a significant barrier with one participant stating: “They are very uneducated on addiction. They have a big stigma when it comes to addicts. If they find out you’re an addict, their whole demeanor changes. They rush you, they slam things, they are very impatient with you and it’s very saddening to see” [17].

#### Lack of motivation or competing priority of drug use

Two of the included studies noted disinterest in testing as a barrier. Barocas et al. [16] noted a ‘lack of motivation’ as a barrier to testing and the study by Ward et al. [14] found that onging drug use was a barrier to engagement in testing since it superceded testing as a priority. Ward et al. investigated the impact of social network interventions and note that the network members had difficulty prioritizing anything outside of avoiding withdrawal, therefore HCV testing engagement was limted; one participant stated “I brought my brother here but he keeps getting high. I brought his girlfriend here but they just keep getting high, man; they don’t want the help” [14].

#### Limited awareness of new treatment options

The paper by Ward et al. [14] described the lack of awareness of treatment options that result in cure and without believing in a cure, participants may not get tested:

I think because [network members] still don’t realize that there is a cure for [HCV]…Because I just recently heard about the cure for it. And when I first heard it, I didn’t want to believe it neither. Because they were always ‘no cure, no cure, no cure’and then all of a sudden somebody said there’s a cure. And so one person said what you’ve been constantly hearing, [that] there’s [no cure], you begin to believe what you hear” [14]

Harris et al. [11] also noted a barrier to be concerns about interferon treatment, which was a poorly tolerated therapy with low efficacy. Tofighi et al. [15] noted the limited awareness of the availability of DAA therapy as a much better tolerated and highly efficacious treatment to be a barrier.

### Enablers to testing

#### Increasing awareness of HCV testing and treatment and providing positive narratives surrounding care

Increasing awareness of cure helped to engage PWID. One participant in the study by Coupland et al. stated that their knowledge of HCV and the treatment has changed: “[What was your understanding?] When you got it [HCV], you got it, that’s it. And you’ll eventually die from it. [But now?] Yeah, I know you can clear it and you can live a normal life” [12].

Ward et al. noted that social networks could help to increase awareness of HCV testing since there was an overwhelming willingness of the participants to discuss HCV testing and treatment in their social networks with people whom they may not know well but have injected drugs with. Many of the participants felt comfortable discussing HCV and many had already had conversations with network members about HCV [14]. One participant stated:

We’re already doing something we have in common…I’m talking about something positive, like going to the program and going to groups and meeting therapists and stuff like that. I would feel more comfortable about, ‘Hey, man, you ever had your Hep C checked out? Because you know we do a lot of damage to our bodies, man, when we are out here using.’ That’s how I would approach it” [14].

This was echoed by another participant who stated:

Hire me. I would [encourage people to get tested and treated for HCV]. I mean, it would give me something to do. It would make me feel better… Because I would only be doing it for so many hours. And you have to go in certain neighborhoods too. That’s another thing. Where there is drug use. And who’s best to go but an ex-addict [14].

Traditionally, negative stories have dissuaded clients from seeking treatment but positive word of mouth stories have made a difference in encouraging people to get tested. Stories of newer more effective and better tolerated therapies, convenient dry-blood-spot testing (which uses a fingerstick blood sample versus having to take a venous blood sample), and Fibroscan® technology to assess liver damage instead of having to get a liver biopsy, have all facilitated engaging participants in care [13]. One participant spoke about the new treatment options: “It’s just general, you just sit and talk, but everybody’s raving about this new stuff, everybody claims that ‘oh I know someone that’s done it’” [13].

#### Positive rapport with provider

Coupland et al. noted the high level of rapport with staff at the community outreach center made a significant difference in engaging PWID;

It’s cool to come and talk to youse and hang out whereas a doctor does not have the same rapport that you would get with me or anybody else. The doctor would go ‘Oh yeah, you’ve got it, this is what you can do, okay see ya later.’ Whereas you care a little bit more [12].

Having well informed and non-judgemental care providers helped as well: “Youse are young and, you know, up with shit. That helps too. Youse have got knowledge of how things are in the streets and what not, because of what youse do” and “There’s no judgment here. You are very understanding and easy going” [8]. Latham et al. found that having site staff that “deal with [drugs and related issues] every day” was important as it meant that they were “not judgemental” [18]. This theme was repeated in research by Phillips et al.:

I mean, I have to say I think [HCV Nurse] is one of the main people behind and she’s, she’s so friendly and nice that she just puts you at ease anyway. There’s not like, you’re not dealing with fearful doctors with a sense of impending doom on all sides [13].

Having providers who are invested in each individual was also helpful, one participant in the study by Harris et al. (2018) stated:

She’s even phoned me up to say, listen, don’t forget, if you don’t want to go, let me know. She’s good enough, she could have just sent me a letter out and just said, well, I sent him a letter, he never turned up [11].

#### Accessible testing options

Accessibility of testing was important. Barocas et al. [16] noted free testing, access to transportation, and mobile testing centers/syringe exchange programs are all enablers to testing. Ward et al. [14] noted that reaching people in their community was helpful. This was echoed in research by Tofighi et al.;

“I am not gonna go to no building and get tested [for HCV] because I don't have the time for that. If that mobile van is sitting out there, and they telling me that's what it's for, I will go in there. It will ease my consciousness” [15].

#### Individualized care tailored to the patient’s preferences and priorities

Choice around testing options is important. Latham et al. found that participants had discrepant views about the type of sample collection used for testing. Some participants preferred a mouth swab as a method of testing: “It’s like less hassle getting blood sounds really intense, but doing a mouth swab, sounds really non-chalant. I’d come every week if that’s all that it was” [18] while another stated:

I’d rather just do the blood work [from a vein]. Cause I’m not just worried about hep C. I’m worried about the whole lot. So I’d rather do the blood ‘cause then I’ll know I haven’t got hep C, hep B and HIV” [18].

Within the study by Latham et al. there were discrepant views regarding the importance of having rapid return of result return as well; most preferred a same-day result whereas for others the same-day result was unnecessary. Those that preferred the same-day result noted that it “saves a lot of stress” if the result is negative and “get[s] the ball rolling sooner rather than later” if the result was positive [18]. Ohers found it unnecessary to get a result in the same day:

I don’t do things like share with other people, give my blood to other people, make other people vulnerable to it, so I don’t have to worry… That’s why it doesn’t matter to me if they give me the result today or next week, whatever” [18].

Coupland et al. noted that making information about HCV testing and treatment salient and tailored to the individual also helped with engagement in testing [12]. In this study the participants were provided information about the importance of HCV testing that was specific to them, in one case a participant resonated with the discussion surrounding risk of transmission to their children:

The part about my kids really stuck out, ‘cause that was the part that was important to me most of all. And you answered my questions regarding that. I think a doctor would have jabbered on a bit. You answered my questions then you went ‘And this is the other parts which could affect your life’. When you gave me the result, you answered my questions first and then you gave the spiel afterwards [12]

## Discussion

The purpose of this scoping review was to explore the qualitative data that exists in the literature regarding the barriers and enablers to HCV testing as experienced by PWID. There are several quantitative studies that provide insight into the barriers and enablers to testing for HCV in this population, however, there are a limited number of studies which explore qualitative data. Qualitative research produces rich, detailed and valid process data based on the participant’s perspectives and interpretation to give a more in-depth understanding of the barriers and enablers [19]. Qualitative data would provide further insight into the barriers and enablers that affect testing for HCV in PWID that the quantitative research may not provide.

This scoping review identified nine studies that provided some data; however, this information was gained through studying other objectives and was not the primary objective of the studies included in this review. Thus, the evidence available to date does not provide a comprehensive understanding of the barriers and enablers to testing as none of the studies set out to specifically explore the perceptions of PWID around HCV testing, though some common themes were identified across studies.

All studies were recent, having been published in the past decade with the majority in the past 5 years. This coincides with the WHO setting goal of eliminating HCV in 2016 and acknowledges that injection drug use is a major contributor to the number of people newly infected and unaware of their HCV status. However, all of the studies were set in large cities such as New York, Boston, and Sydney, which raises the question as to whether the experiences of PWID in these large urban areas is relevant to those who live in smaller centers or rural areas. There could be differences due to the vast geography and testing may be inaccessible in rural areas due to lack of providers or lack of hospital settings. There are known disparities in health and healthcare of rural residents compared to non-rural residents in many areas; rural patients are seen to have less access to healthcare resources as well as more concerns over confidentiality, and may experience more embarrassment around stigmatizing illnesses [20]. There may also be a difference between perceptions of PWID living rural vs non-rural settings.

Common barriers and enablers were seen across all the studies and highlight the important factors that should be considered when designing a testing program to effectively engage PWID. Increasing awareness of testing options, HCV transmission and risk; educating and training care providers to be non-judgemental; and providing accessible options in the community will all be important factors based on this review.

The setting of the studies varied and thus participants in each study may have different motivations or variables in their lives that could impact the barriers they experience and their view of enablers. Although multiple studies are set within the community, either through community engagement or needle syringe exchange programs, Tofighi et al. [15] interviewed PWID in an inpatient detoxification setting. The experiences of these clients may be different since they are enrolled in detoxification and may not intend to continue to inject drugs, as is the case in some of the other studies. Phillips et al. [13] interviewed participants accessing drug and alcohol treatment services and thus may be more engaged in care than those that are not linked to services. Harris et al. [10]and Harris et al. [11] both interviewed participants of opioid substitution therapy services (OST) and drug therapy services (DTS) and thus they also portray views and experiences that may differ from PWID who have no intention of stopping their drug use. Those PWID that are engaged in care through OST or DTS may be more likely to have addressed some of the barriers to care such as lack of education, lack of rapport with a provider, accessibility of testing options, etc. by availing of these services. PWID who do not access these services may experience more of these barriers.

Some of the studies included interviews with PWID who are already diagnosed with HCV [12–14, 17] and others include both PWID not previously diagnosed and PWID diagnosed with HCV. This may affect what is seen as barriers and enablers as the participants recall their own experiences differently based on their HCV status and engagement in HCV care. Among the studies which included participants that had known HCV positive status a common barrier was lack of support for testing and treatment and the negative discourse around HCV among PWID [12–14, 17]. Even though these studies were recent (2018–2021) and within the era of DAA therapies the participants may not have known about the availability of these therapies or offered treatment with these therapies.

The age range in many of the included studies was broad but Skeer et al. [17] looked only at the experiences of young people aged 15–30; the testing experience may be different in this specific demographic. The majority of the participants in each study were male with the exception of Skeer et al. [17] which had an equal representation from male and females. Perspectives of PWID from various gender, and sexual identity backgrounds is needed to fully understand these barriers and enablers in other populations. Most of the studies had a majority Caucasian population with the exception of Ward et al. [14] and Tofighi et al. [15] Additional research from the perspectives of black, indigenous, and other people of colour is needed.

HCV testing options are expanding and point-of-care screening for HCV is available as an option in Canada. The OraQuick Rapid Antibody Test® approved by Health Canada detects antibodies to HCV with a 95.9% sensitivity and a 99% specificity [21]. This option involves a finger-prick blood sample which is tested immediately on site and can provide results within 20 min [22]. A positive antibody test means that the person has had HCV at some point in their lifetime and a confirmatory test is required to determine whether the person has a current HCV infection. A confirmatory test detects viral RNA in the blood and can currently only be done through a venipuncture blood sample and testing in a lab. There are currently no HCV RNA point-of-care tests approved in Canada but there is one being used for research purposes in Canada today which may be licensed in future. The evolution of testing opportunities may help to address some of the barriers and enablers that have been highlighted by this scoping review, however, point-of-care testing is not reliably funded in any jurisdiction in Canada, making this another largely inaccessible testing option [23]. If this option was more widely available it would allow PWID to choose this method of sample collection (finger-stick versus venipuncture) and have the option of a screening test that would provide a quicker result if this was of interest to them. This echoes the enabling factors seen in this scoping review of ‘ accessible testing options’ and ‘individualized care tailored to the person’s preferences and priorities”. As testing continues to evolve the potential for an approved HCV RNA point-of-care test would further enhance testing by removing the need for a confirmatory laboratory test and make testing even more accessible.

The barriers and enablers explored in this scoping review are from the perspective of PWID and these factors may be quite different from those from the healthcare provider perspective. Literature shows that providers experience system level barriers (lack of infrastructure and lack of bureaucratic support and multiple competing professional responsibilities), limited knowledge of HCV and being reticent to treat HCV patients with current drug use and psychiatric needs [24]. These provider-level barriers and enablers will also impact PWID and should be considered when developing testing programs for this population. Another structural barrier that are was not addressed in the studies in this scoping review could be the cost of the test. In Canada, visits to a healthcare provider and subsequent testing for HCV would be free of charge and thus cost will likely not be a factor, however, in other countries where people are required to pay for these services there would likely be significant impact on testing in the PWID population.

One setting for HCV testing that was not explored in this scoping review is pharmacy-based testing. In recent years, a pharmacy-based testing model has been proposed to address barriers to testing for sexually transmitted and blood-borne infections (STBBI). The APPROACH pilot study [25] found that pharmacy-based point-of-care testing for HIV was feasible and highly acceptable to participants and pharmacists. The success of this program led to the APPROACH 2.0 study which launched in December 2022 and builds on the pilot to expand testing to also include HCV and syphilis testing [26]. The APPROACH study team hypothesized that a pharmacy-based testing program could overcome some of the barriers that have been identified in this scoping review [25]. For example, pharmacy-based testing could be seen as an accessible option in the community since pharmacies are widely available throughout many geographical areas. Pharmacy-based testing, which offers point-of-care or dry blood spot testing, could offer a solution to those who may not want to be tested via a blood sample drawn from a vein. A recent study by Klepser et al. screened 1164 patients at increased risk for HIV, HCV, or both (including people who inject drugs) at 61 participating pharmacies and concluded that patients at risk of HIV or HCV can benefit from screening at community pharmacies [27], however, the factors that attracted people to testing in this venue are not explored in this quantitative study. Another study by Radley et al. concluded that using pharmacists to deliver an HCV care pathway made testing more accessible and improved engagement but only provided quantitative data, not exploring the reasons behind engagement in this testing venue [28]. Pharmacy based testing may address stigma as a barrier if PWID see the pharmacy as a discrete place for testing, as pharmacies offer many various services, however, some patients may experience healthcare related stigma at pharmacies and may see this as a barrier. There have been quantitative studies exploring HCV testing in community pharmacies that have shown that testing is accessible and effective, but these factors are not explored in detail [27, 28]. To our knowledge there has been no qualitative research done to specifically assess the perceptions of a pharmacy-based testing program in PWID, therefore, qualitative research would help provide more insight into barriers and facilitators to HCV-testing uptake.

This scoping review has some notable limitations. The search only included those papers published in the English language and therefore other countries’ experiences may have been missed. Grey literature was not included and therefore some additional data may have been missed. This scoping review included only those who inject drugs and not those who use drugs by other means (i.e. oral or inhaled) though these behaviors have also been associated with increased risk of HCV infection.

## Conclusion

This scoping review determined that there has been some research in this area over the past decade that has shown some common barriers and enablers to testing for HCV among PWID though it has not been focused to specifically address this research question. More research is needed on the preferences of PWID regarding accessing HCV testing services in smaller centers and rural areas which may have different healthcare access and programming. As the field of HCV diagnostics is rapidly advancing, future research which explores barriers and enablers to these new testing technologies from the PWID perspective will be critical to meeting the aims of the World Health Organization elimination targets.

## Supplementary Information

**Additional file 1.** Search Strategy.

## Data Availability

All data generated or analyzed during this study are included in this published article and its supplementary files.

## Abbreviations

PWID: People Who Inject Drugs
HCV: Hepatitis C Virus
HIV: Human Immunodeficiency Virus
WHO: World Health Organization
