# Supplementary material for: Barriers and enablers to testing for hepatitis C virus infection in people who inject drugs – a scoping review of the qualitative evidence
Source: BMC Public Health. 2023 Jun 1;23:1038. doi: 10.1186/s12889-023-16017-8 (PMC10234098; doi:10.1186/s12889-023-16017-8)
Supplement: Supplementary file 1 — Additional file 1. Search Strategy. [file 12889_2023_16017_MOESM1_ESM.docx]

**Appendix A: Search Strategy**

1 substance abuse, intravenous/ or exp Drug Users/ or Buprenorphine, Naloxone Drug Combination/tu or exp Methadone/tu or Opiate Substitution Treatment/ 25813

2 ((methadone or suboxone or opiate or opioid) adj2 (treatment or therapy or therapeutic or clinic)).tw,kf. 13219

3 ((people or person or persons or women or woman or men or man) adj2 (inject* or "use" or uses or using or used or usage or addict* or depend* or misus* or abus*) adj (drugs or drug or substance or substances or heroin* or dope or 'china white' or smack or cocaine or blow or bump or coke or rock or snow or toot or crack or meth or Molly or MDMA or crank or ecstasy or flake or Krokodil or crocodil or methamphetamine* or fentanyl or ketamine or DMT or PCP or barbiturate* or opiate* or opioid* or narcotic* or 'bath salts')).tw,kf. 5094

4 (pwid or pwud).tw,kf. 2182

5 ((drugs or drug or substance or substances or heroin* or dope or 'china white' or smack or cocaine or blow or bump or coke or rock or snow or toot or crack or meth or Molly or MDMA or crank or ecstasy or flake or Krokodil or crocodil or methamphetamine* or fentanyl or ketamine or DMT or PCP or barbiturate* or opiate* or opioid* or narcotic* or 'bath salts') and (inject* or intravenous or IV)).tw,kf. 232780

6 ((illicit or illegal or recreational) adj2 (drug or drugs or substance or substances) adj2 ("use" or uses or using or used or usage or addict* or depend* or misus* or abus*)).tw,kf. 10474

7 1 or 2 or 3 or 4 or 5 or 6 262577

8 exp Hepatitis C/di, dg 9602

9 ((test* or screen*) and (Hepatitis C or Hep C or HCV)).tw,kf. 22943

10 (exp Hepacivirus/ or exp Hepatitis C/) and (test* or screen*).tw,kf. 16659

11 (satisf* or percept* or enable* or perspectiv* or attitud* or opinion* or view or views or preferenc* or experienc* or barrier* or facilitat* or motivat* or challeng* or obstacle* or knowledge).tw,kf. 4846690

12 exp Attitude to Health/ 429382

13 exp Patient Satisfaction/ 92138

14 11 or 12 or 13 5035472

15 8 or 9 or 10 29565

16 7 and 14 and 15 979
